# Supplementary material for: Disentangling age, gender, and racial/ethnic disparities in multiple myeloma burden: a modeling study
Source: Nat Commun. 2023 Sep 20;14:5768. doi: 10.1038/s41467-023-41223-8 (PMC10511740; doi:10.1038/s41467-023-41223-8)
Supplement: Supplementary file 1 — Supplementary Information [file 41467_2023_41223_MOESM1_ESM.pdf]

SUPPLEMENTARY INFORMATION

Methods

Data

MM Mortality Rates

To estimate gender-, and race/ethnicity-specific mortality rates in individuals with MM, we fit exponential survival curves to SEER survival data using the method of least squares[1]. The survival data from SEER included individuals of all ages, so we assumed that the mortality rate of MM did not depend upon the age of diagnosis. We report the estimated mortality rate as well as the minimized sum of least squares for each gender- and race/ethnicity- pairing.

Simulation Study

To confirm that our inference framework was capable of correctly estimating each parameter, we performed a simulation study. Because of the wide uniform prior distributions that we placed on each parameter, randomly sampling parameter sets was an inefficient strategy to identify parameter sets that could generate non-zero synthetic data. Accordingly, we adopted a multi-step strategy to select parameter sets for our simulation study. First, we restricted the prior distribution (Table S1). Second, we sampled one million parameter steps from the restricted prior distribution using a Sobol sequence in the *pomp* R package[2]. Finally, for each parameter set, we simulated a synthetic data set using the likelihood as a data-generating process and included the simulation study if the MGUS prevalence was greater than or equal to 1% in at least one age group and the MM incidence was greater than or equal to 1 per 100,000 individuals in at least one age group. These constraints were conservatively chosen as they are far less than the observed MGUS prevalence and MM incidence in the real data, and subjecting the synthetic

datasets to these constraints ensured that there was greater information content in the simulated data to infer the parameter sets. For each parameter set that satisfied the above constraints, we applied five independent chains of our MCMC algorithm to the corresponding synthetic dataset. We then assessed convergence and compared the posterior distribution of each parameter to the true value.

Table S1. Parameter definitions and prior distributions for the simulation study.

Parameter	Definition	Prior
γ_{MGUS}	Intercept for rate of MGUS development	[-20,0]
$\beta_{\mathrm{MGUS},a}$	Age coefficient for rate of MGUS development	[0,0.5]
$\beta_{\mathrm{MGUS},s}$	Gender coefficient for rate of MGUS development	[-2,2]
$\beta_{\mathrm{MGUS},r}$	Race/ethnicity coefficient for rate of MGUS development	[-2,2]
γ_{MM}	Intercept for rate of MM development	[-20,0]
$\beta_{\mathrm{MM},a}$	Age coefficient for rate of MM development	[-2,1]
β_{MM,a^2}	Quadratic age coefficient for rate of MM development	[-0.25,0.25]
$\beta_{\mathrm{MM},s}$	Gender coefficient for rate of MM development	[-2,2]
$\beta_{\mathrm{MM},r}$	Race/ethnicity coefficient for rate of MM development	[-2,2]
τ^2	Variance of MM incidence	[0,1]

Sensitivity Analyses

Assumption of Age Effects for MGUS and MM

In the main analysis, we allowed the rates of progression from healthy to MGUS and from MGUS to MM to depend upon age. To test the validity of this assumption, we fit an alternative model in which the rates of progression from healthy to MGUS and from MGUS to MM did not depend upon age. We then compared this model to the model presented in the main analysis on the basis of the deviance information criterion (DIC)[3].

Assumption of Quadratic Age Term for MM

In the main analysis, we included a quadratic age term in the rate of progression from MGUS to MM, because we observed that the incidence of MM declined in older age groups. To ensure that we were not overfitting to the data, we fit an alternative model in which the rate of progression from MGUS to MM did not include a quadratic age term. We then compared this model to the model presented in the main analysis on the basis of DIC[3].

Assumption of MGUS Mortality

In our main analysis, we followed Therneau et al.[4] and assumed that mortality of MGUS-positive individuals was 1.25 times the baseline age- and race/ethnicity-specific mortality for men and 1.11 times the baseline age- and race/ethnicity-specific mortality for women. To test the sensitivity of our results to this assumption, we fit an alternative model in which the mortality of MGUS-positive individuals was equal to the baseline age-, gender-, and race/ethnicity-specific mortality. We then compared the parameter estimates that we obtained under this alternative model to the parameter estimates that we obtained in the main analysis.

Choice of Prior Distribution

We examined whether the results of our analysis were sensitive to our choice of prior distributions. For all intercept and coefficient parameters, we assumed that the standard deviation of our prior distribution was twice as large as the standard deviation used in our main analysis. Additionally, for the standard deviation σ of the SEER MM incidence, we assumed that the upper bound of the uniform distribution was twice as large as the upper bound used in our main analysis. We fit the model using these prior distributions and compared the parameter estimates to those obtained in the main analysis.

Choice of SEER MM Data

We examined whether the year of SEER MM incidence data affected the conclusions that we reached about the contributions of age, gender, and race/ethnicity to disparities in MM incidence. We compared the estimated quantities obtained in the main analysis using SEER data from 2010 to the estimated quantities obtained in alternative analysis using SEER data from 2004[1]. In this alternative analysis, all data on population and mortality were from 2004, not 2010.

Comparison with Other Cohorts

We comparing our model predictions to data sources that were not used to fit the model. Specifically, we compared predicted MGUS prevalence by age and gender from our model to predicted MGUS prevalence by age and gender in Olmsted County, Minnesota between 1995 and 2001[5]. Because 97.3% of the Olmsted County cohort identified as white, we made use of model predictions for non-Hispanic white men and women. Next, we compared our model's prediction for lifetime risk of developing MM to estimates reported by the American Cancer Society's Cancer Statistics from 2023[6]. Estimates were only available by gender, so we

compared our model predictions for non-Hispanic white people, non-Hispanic Black people, and a composite of non-Hispanic white people and non-Hispanic Black people weighted by population size.

Results

MM Mortality Rates

Figure S1 shows fitted survivorship as a function of the number of years since diagnosis for individuals with MM. For non-Hispanic white men, the estimated MM mortality rate was 0.13 yr^{-1}, corresponding to a mean time from diagnosis to death of 7.4 years. For non-Hispanic white women, the estimated MM mortality rate was 0.14 yr^{-1}, corresponding to a mean time from diagnosis to death of 7.3 years. For non-Hispanic Black men, the estimated MM mortality rate was 0.13 yr^{-1}, corresponding to a mean time from diagnosis to death of 7.9 years. For non-Hispanic Black women, the estimated MM mortality rate was 0.12 yr^{-1}, corresponding to a mean time from diagnosis to death of 8.1 years.

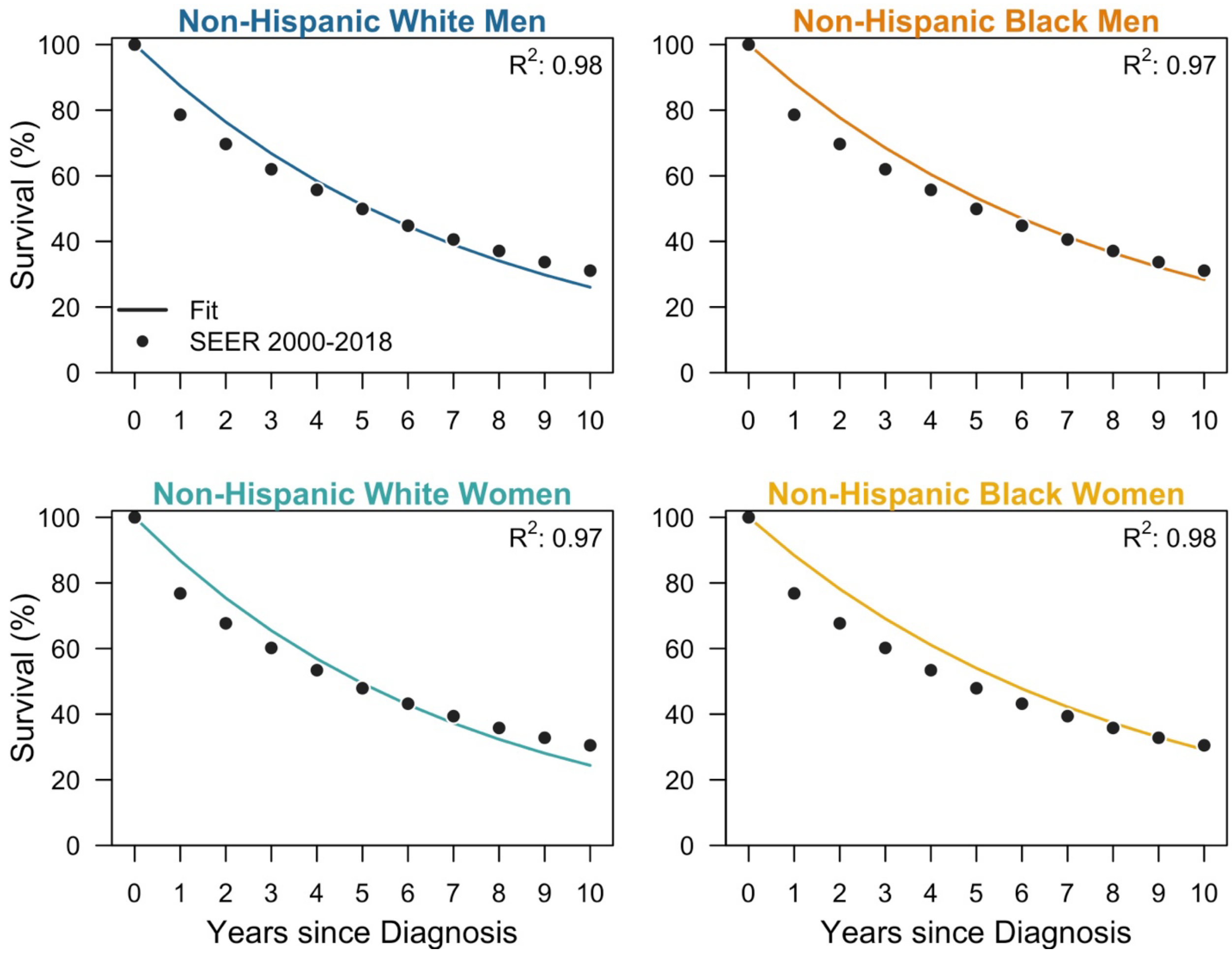

Figure S1. Fitted multiple myeloma survivorship by gender and race/ethnicity. The fitted survivorship curves (lines) as a function of the years since multiple myeloma diagnosis are shown for non-Hispanic white men (dark blue), non-Hispanic white women (light blue), non-Hispanic Black men (dark orange), and non-Hispanic Black women (light orange). Fitted survivorship curves are compared to the respective SEER 2000-2018 survivorship data (points) to each curve was fitted. The R^2 is reported for each fit.

Simulation Study

From the one million parameters that we sampled from the prior distribution, we identified 410 parameter sets that generated MGUS prevalence of at least 1% in at least one age group and MM

incidence of at least 1 per 100,000 individuals in at least one age group. We then applied our MCMC algorithm to synthetic data sets generated from each of these 410 parameter sets and assessed convergence of five independent chains using the Gelman-Rubin statistics. We found statistical support for convergence in our inferences on 229 of the 410 synthetic data sets.

For each parameter, we observed strong agreement between the inferred values and true values with appropriate levels of uncertainty (Fig. S2). The coverage probabilities, defined as the proportion of data sets for which the true value falls within the 95% credible intervals, ranged from 0.92 for τ^2 to 0.97 for β_{MM,a^2} with a mean of 0.95 across all ten parameters. That the coverage probabilities fell at or near 0.95 indicates that our inference algorithm is well-calibrated.

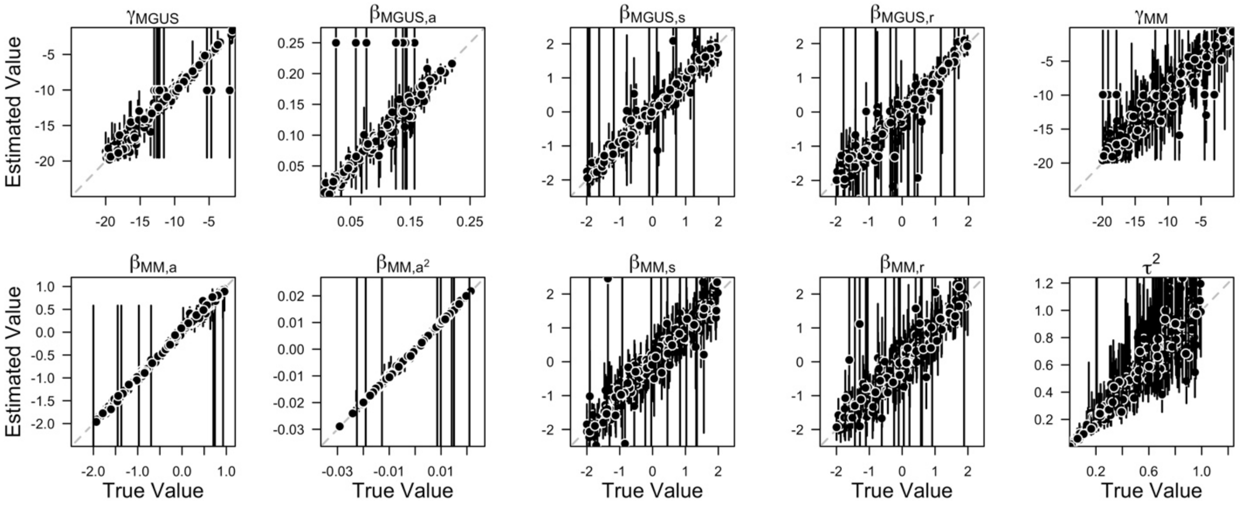

Figure S2. Comparison of inferred parameter estimates to true parameter values. The inferred parameter estimates (vertical axis) are compared to the true parameter value (horizontal axis), where estimates that fall along the one-to-one line indicate greater agreement with the true value. Points are the posterior median of the parameter in each simulated dataset, and the vertical

line segments denote the corresponding 95% credible interval. Each 95% credible interval was calculated from n = 50,010 posterior samples.

Inference on NHANES and SEER Data

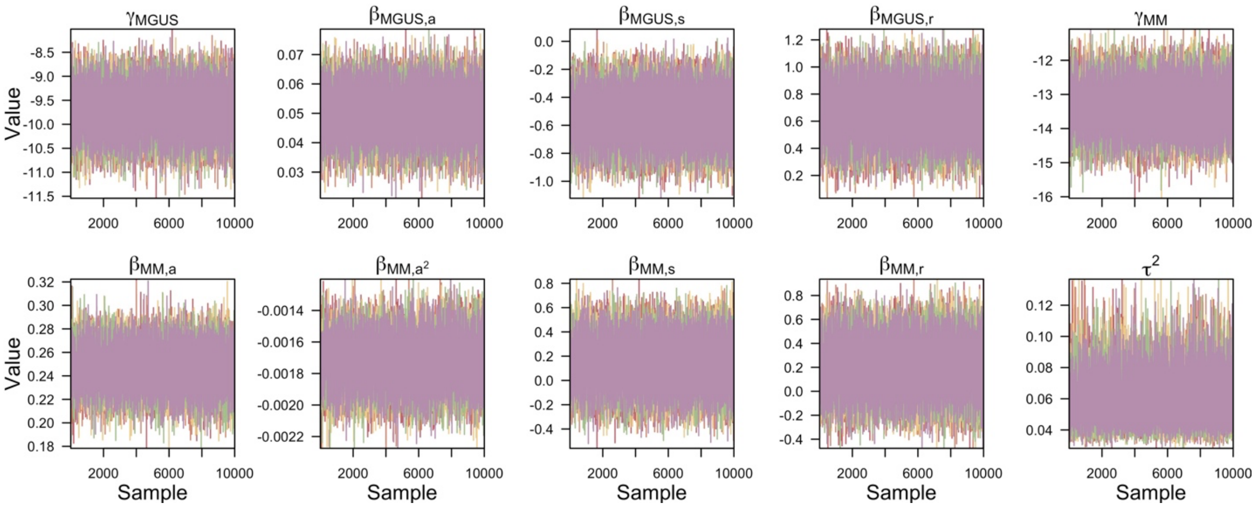

Figure S3. Posterior traces of the estimated parameters. The traces for each estimated parameter are shown. Each color represents one of five independent chains that were run to assess convergence.

Table S2. Parameter estimates and convergence statistics.

Parameter	Median Estimate (95% CI)	Gelman-Rubin Statistic
γ_{MGUS}	-9.7 (-11.0 – -8.9)	1.0
$\beta_{\mathrm{MGUS},a}$	0.051 (0.036 – 0.065)	1.0
$\beta_{\mathrm{MGUS},s}$	-0.54 (-0.84 – -0.24)	1.0
$\beta_{\mathrm{MGUS},r}$	0.68 (0.34 – 1.00)	1.0
γ_{MM}	-13 (-15 – -12)	1.0

$\beta_{\mathrm{MM},a}$	0.25 (0.22 – 0.28)	1.0
β_{MM,a^2}	-0.0017 (-0.0020 – -0.0015)	1.0
$\beta_{\mathrm{MM},s}$	0.14 (-0.20 – 0.49)	1.0
$\beta_{\mathrm{MM},r}$	0.20 (-0.17 – 0.58)	1.0
τ^2	0.058 (0.39 – 0.092)	1.0

Sensitivity Analyses

Assumption of Age Effects for MGUS and MM

We fit an alternative model in which the rates of progression from healthy to MGUS and from MGUS to MM did not depend upon age and compared this alternative model to the model used in the main analysis on the basis of DIC. After assessing convergence (Table S3), we found that the DIC of the alternative model, we found that the DIC of the alternative model was 340, compared to a DIC of 146 for the model in the main analysis. That the difference in the DIC was much greater than 10 provides statistical support for an age-dependent effect on the rate of progression from healthy to MGUS and from MGUS to MM.

Table S3. Parameter estimates and convergence statistics for age-independent model

Parameter	Median Estimate (95% CI)	Gelman-Rubin Statistic
γ_{MGUS}	-7.4 (-7.6 – -7.2)	1.0
$\beta_{\mathrm{MGUS},s}$	-0.47 (-0.79– -0.16)	1.0
$\beta_{\mathrm{MGUS},r}$	0.53 (0.19 – 0.86)	1.0
γ_{MM}	-5.8 (-6.6 – -5.1)	1.0

$\beta_{\mathrm{MM},s}$	0.049 (-0.83 – 0.92)	1.0
$\beta_{\mathrm{MM},r}$	0.35 (-0.53 – 1.2)	1.0
τ^2	2.2 (1.5 – 3.5)	1.0

Assumption of Quadratic Age Term for MM

We fit an alternative model in which the rate of progression from MGUS to MM did not contain a quadratic age term and then compared this alternative model to the model in the main analysis on the basis of DIC. After assessing convergence (Table S4), we found that the DIC of the alternative model was 225, compared to a DIC of 146 for the model in the main analysis. That the difference in the DIC was much greater than 10 provides statistical support for the model used in the main analysis.

Table S4. Parameter estimates and convergence statistics for non-quadratic age model

Parameter	Median Estimate (95% CI)	Gelman-Rubin Statistic
γ_{MGUS}	-9.4 (-10.0 – -8.5)	1.0
$\beta_{\mathrm{MGUS},a}$	0.045 (0.029 – 0.060)	1.0
$\beta_{\mathrm{MGUS},s}$	-0.51 (-0.82 – -0.20)	1.0
$\beta_{\mathrm{MGUS},r}$	0.64 (0.29 – 0.97)	1.0
γ_{MM}	-8 (-9.3 – -6.8)	1.0
$\beta_{\mathrm{MM},a}$	0.044 (0.027 – 0.062)	1.0
$\beta_{\mathrm{MM},s}$	0.10 (-0.35 – 0.56)	1.0

$\beta_{MM,r}$	0.24 (-0.24 – 0.72)	1.0
τ^2	0.31 (0.20 – 0.49)	1.0

Assumption of MGUS Mortality

To test the sensitivity of our analysis to the assumption of MGUS mortality, we compared our parameter estimates from our primary analysis to those obtained from an alternative analysis in which we assumed that there was no relative difference in mortality among MGUS-positive individuals as compared to the baseline age-, gender-, and race/ethnicity-stratified mortality. After assessing convergence, we found that our parameter estimates were robust to our assumption of MGUS mortality (Fig. S4). The 95% credible intervals overlapped for each of the parameters, indicating that we found no statistically significant difference in their estimate for this assumption.

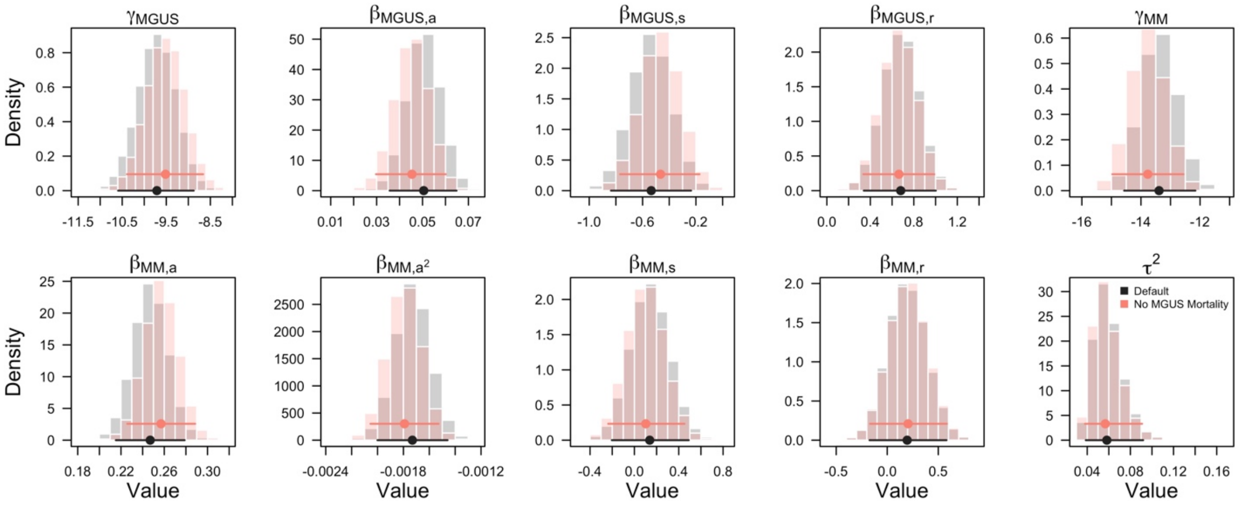

Figure S4. Sensitivity of the posterior distribution to assumption of MGUS mortality. The marginal posterior distributions for each parameter are shown under the default assumption (black) in which MGUS mortality for men and women are multiples estimated from Therneau et

al. (2012) of all-cause mortality and the alternative assumption in which there is no relative increase in MGUS mortality (orange). The posterior median (circle) and the 95% credible intervals (horizontal segments) are shown for both sets of assumptions. Each 95% credible interval was calculated from n = 50,010 posterior samples.

Choice of Prior Distribution

To test whether the choice of prior distribution influenced the parameter estimates that we obtained, we compared the parameter estimates from the primary analysis to those obtained from an alternative analysis in which we widened the prior distribution for each parameter. After assessing convergence, we found that the posterior medians and 95% credible intervals overlapped for each parameter, suggesting that our results were robust to our choice of prior distribution (Fig. S5).

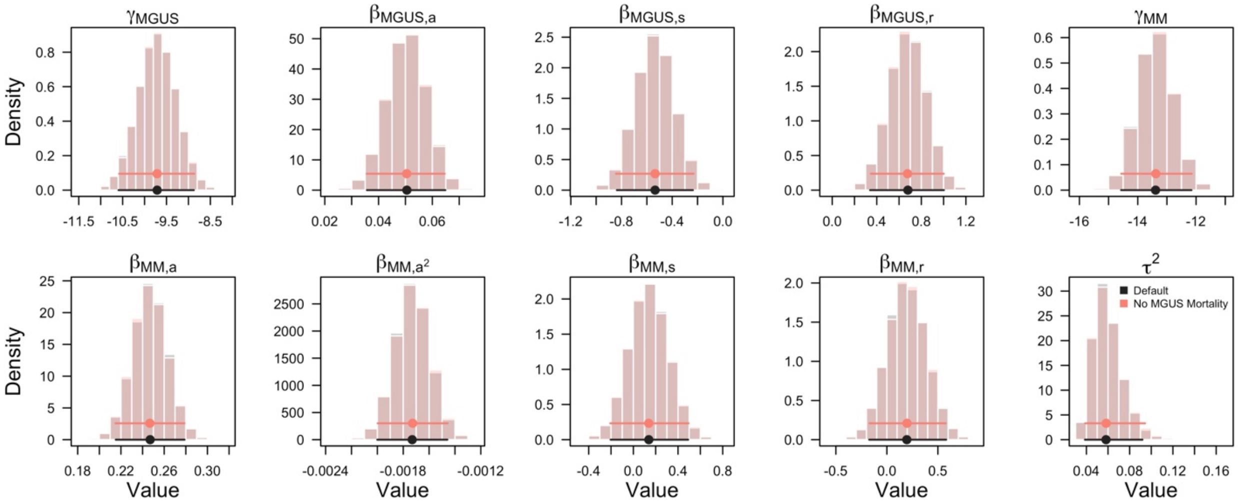

Figure S5. Sensitivity of the posterior distribution to the choice of prior distribution. The marginal posterior distributions for each parameter are shown under the default prior distribution (black) and the alternative assumption in which each uniform prior distribution was twice as

wide (orange). The posterior median (circle) and the 95% credible intervals (horizontal segments) are shown for both sets of assumptions. Each 95% credible interval was calculated from n = 50,010 posterior samples.

Choice of SEER MM Data

To evaluate whether the year of the SEER MM incidence affected the conclusions of our analysis, we fit our model to 2004 MM incidence and compared its parameter estimates to the parameter estimates from the model fit to 2010 MM incidence. Both models converged upon their respective posterior distributions, and the Gelman-Rubin statistics for all parameters were 1.0.

The parameter estimates were robust to the year of MM incidence (Table S5). The coefficient, $\beta_{\mathrm{MM},r}$, for the effect of race/ethnicity on the progression from MGUS to MM was higher when using 2004 MM incidence data compared to 2010 MM incidence data, though the 95% credible intervals overlapped. Importantly, both 95% credible intervals for $\beta_{\mathrm{MM},r}$ included zero, indicating that the race/ethnicity did not affect the progression from MGUS to MM. That the parameter estimates were largely robust to the year of MM incidence supports the conclusions that we reached in the main analysis.

Table S5. Comparison of the parameter estimates from models fit to 2004 and 2010 SEER data.

**Parameter**	**Median Estimate (95% CI)**	
	**2004 SEER**	**2010 SEER**
γ_{MGUS}	-9.7 (-11.0 – -8.9)	-9.7 (-11.0 – -8.9)

$\beta_{\text{MGUS},a}$	0.050 (0.036 – 0.064)	0.051 (0.036 – 0.065)
$\beta_{\text{MGUS},s}$	-0.54 (-0.85 – -0.24)	-0.54 (-0.84 – -0.24)
$\beta_{\text{MGUS},r}$	0.65 (0.31 – 0.98)	0.68 (0.34 – 1.00)
γ_{MM}	-13 (-14 – -12)	-13 (-15 – -12)
$\beta_{\text{MM},a}$	0.22 (0.20 – 0.25)	0.25 (0.22 – 0.28)
β_{MM,a^2}	-0.0015 (-0.0018 – -0.0013)	-0.0017 (-0.0020 – -0.0015)
$\beta_{\text{MM},s}$	0.13 (-0.21 – 0.48)	0.14 (-0.20 – 0.49)
$\beta_{\text{MM},r}$	0.33 (-0.042 – 0.72)	0.20 (-0.17 – 0.58)
τ^2	0.044 (0.029 – 0.070)	0.058 (0.39 – 0.092)

Comparison with Other Cohorts

We compared model estimates of MGUS prevalence for non-Hispanic white men and women to estimates from Olmsted County, Minnesota between 1995-2001[5]. Although the data used for validation reflects a single cohort from a separate time period in one geographical location, there is reasonably good agreement between the model predictions and the validation data with respect to the magnitude of MGUS prevalence and its relationship with age (Fig. S6). A previous study comparing NHANES 1999-2003 to the Olmsted County cohort noted higher MGUS prevalence in Olmsted County, suggesting that the higher MGUS prevalence in Olmsted County may reflect geographical variation[7].

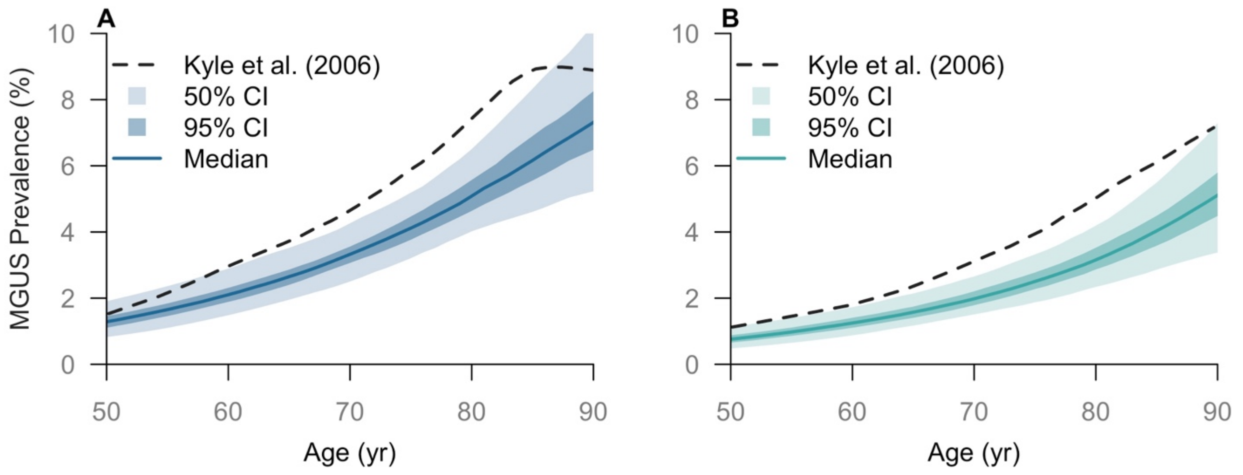

Figure S6. Comparison of MGUS prevalence for non-Hispanic white men and women. Model predictions of MGUS prevalence for non-Hispanic white (A) men and (B) women are shown as a function of age and compared to estimates from Kyle et al.[5] in Olmsted County, Minnesota. The dashed black line is the estimate from Kyle et al.[5]. The solid line is median posterior model prediction, the darker shaded area is the 50% credible interval (CI), and the lighter shaded area is the 95% CI. Each 95% credible interval was calculated from n = 50,010 posterior samples.

We additionally compared our model estimates of lifetime risk of developing MM to estimates published by the American Cancer Society[6]. Reported lifetime risk of developing MM for men is 0.9%. Our model predictions for the lifetime risk of developing MM were 0.77% (95% CI: 0.68 – 0.87%) for non-Hispanic white men and 1.42% (95% CI: 1.25 – 1.59%) for non-Hispanic Black men. Weighting each race/ethnicity by population size, we obtained a lifetime risk of developing MM for males of 0.88% (95% CI: 0.77 – 0.99%), comparable to the estimate reported by the American Cancer Society. For women, reported lifetime risk of developing MM is 0.7%. Our model predictions for the lifetime risk of developing MM were 0.66% (95% CI: 0.57 – 0.75%) for non-Hispanic white women and 1.33% (95% CI: 1.18 – 1.48%) for non-

Hispanic Black women. Weighting each race/ethnicity by population size, we obtained a lifetime risk of developing MM for women of 0.77% (95% CI: 0.68 – 0.87%), comparable to the estimate reported by the American Cancer Society.

REFERENCES

1. National Cancer Institute. Surveillance, Epidemiology, and End Results (SEER) Program (www.seer.cancer.gov) SEER*Stat Database: Incidence - SEER Research Data, Nov 2021 Sub (1975-2019) - Linked To County Attributes - Time Dependent (1990-2019) Income/Rurality, 1969-2020 Counties. (2022).
2. King, A. A., Nguyen, D. & Ionides, E. L. Statistical Inference for Partially Observed Markov Processes via the R Package pomp. (2015) doi:10.48550/ARXIV.1509.00503.
3. Spiegelhalter, D. J., Best, N. G., Carlin, B. P. & van der Linde, A. Bayesian measures of model complexity and fit. *J. R. Stat. Soc. Ser. B Stat. Methodol.* **64**, 583–639 (2002).
4. Therneau, T. M. *et al.* Incidence of Monoclonal Gammopathy of Undetermined Significance and Estimation of Duration Before First Clinical Recognition. *Mayo Clin. Proc.* **87**, 1071–1079 (2012).
5. Kyle, R. A. *et al.* Prevalence of Monoclonal Gammopathy of Undetermined Significance. *N. Engl. J. Med.* **354**, 1362–1369 (2006).
6. Siegel, R. L., Miller, K. D., Wagle, N. S. & Jemal, A. Cancer statistics, 2023. *CA. Cancer J. Clin.* **73**, 17–48 (2023).
7. Landgren, O. *et al.* Racial disparities in the prevalence of monoclonal gammopathies: a population-based study of 12 482 persons from the National Health and Nutritional Examination Survey. *Leukemia* **28**, 1537–1542 (2014).
