# Supplementary material for: Disentangling age, gender, and racial/ethnic disparities in multiple myeloma burden: a modeling study
Source: Nat Commun. 2023 Sep 20;14:5768. doi: 10.1038/s41467-023-41223-8 (PMC10511740; doi:10.1038/s41467-023-41223-8)
Supplement: Supplementary file 3 — Reporting Summary [file 41467_2023_41223_MOESM3_ESM.pdf]

nature portfolio

Corresponding author(s): John Huber

Last updated by author(s): Aug 8, 2023

Reporting Summary

Nature Portfolio wishes to improve the reproducibility of the work that we publish. This form provides structure for consistency and transparency in reporting. For further information on Nature Portfolio policies, see our Editorial Policies and the Editorial Policy Checklist.

Statistics

For all statistical analyses, confirm that the following items are present in the figure legend, table legend, main text, or Methods section.

n/a	Confirmed	
☐	☒	The exact sample size (*n*) for each experimental group/condition, given as a discrete number and unit of measurement
☐	☒	A statement on whether measurements were taken from distinct samples or whether the same sample was measured repeatedly
☒	☐	The statistical test(s) used AND whether they are one- or two-sided<br>*Only common tests should be described solely by name; describe more complex techniques in the Methods section.*
☐	☒	A description of all covariates tested
☐	☒	A description of any assumptions or corrections, such as tests of normality and adjustment for multiple comparisons
☐	☒	A full description of the statistical parameters including central tendency (e.g. means) or other basic estimates (e.g. regression coefficient) AND variation (e.g. standard deviation) or associated estimates of uncertainty (e.g. confidence intervals)
☒	☐	For null hypothesis testing, the test statistic (e.g. *F*, *t*, *r*) with confidence intervals, effect sizes, degrees of freedom and *P* value noted<br>*Give P values as exact values whenever suitable.*
☐	☒	For Bayesian analysis, information on the choice of priors and Markov chain Monte Carlo settings
☒	☐	For hierarchical and complex designs, identification of the appropriate level for tests and full reporting of outcomes
☒	☐	Estimates of effect sizes (e.g. Cohen's *d*, Pearson's *r*), indicating how they were calculated

Our web collection on statistics for biologists contains articles on many of the points above.

Software and code

Policy information about availability of computer code

Data collection	SEER*Stat was used to obtain MM incidence data from SEER.
Data analysis	All data and code to reproduce the analyses can be found at https://github.com/johnhhuber/MM_Model_Race. Analyses were conducted in R using the BayesianTools and pomp R packages

For manuscripts utilizing custom algorithms or software that are central to the research but not yet described in published literature, software must be made available to editors and reviewers. We strongly encourage code deposition in a community repository (e.g. GitHub). See the Nature Portfolio guidelines for submitting code & software for further information.

Data

Policy information about availability of data

All manuscripts must include a data availability statement. This statement should provide the following information, where applicable:

- Accession codes, unique identifiers, or web links for publicly available datasets
- A description of any restrictions on data availability
- For clinical datasets or third party data, please ensure that the statement adheres to our policy

Databases used: National Health and Nutritional Examination Survey (NHANES) 1999-2004 (https://www.cdc.gov/nchs/nhanes/index.htm); CDC Life Tables (https://www.cdc.gov/nchs/products/life_tables.htm); CDC Wonder Population Database (https://wonder.cdc.gov/Bridged-Race-v2020.HTML); SEER*STAT Cancer Incidence (https://seer.cancer.gov/seerstat/); All data used in this analysis can be found at https://github.com/johnhhuber/MM_Model_Race

Research involving human participants, their data, or biological material

Policy information about studies with human participants or human data. See also policy information about sex, gender (identity/presentation), and sexual orientation and race, ethnicity and racism.

Reporting on sex and gender	Self-reported gender was obtained from NHANES, CDC Wonder Population Database, and SEER
Reporting on race, ethnicity, or other socially relevant groupings	Self-reported race/ethnicity was obtained from NHANES, CDC Wonder Population Database, and SEER.
Population characteristics	Age, gender, and race/ethnicity are reported for all data included in this study. In addition, MGUS diagnosis is reported for NHANES.
Recruitment	This is a secondary data analysis of publicly available data.
Ethics oversight	Washington University in St. Louis

Note that full information on the approval of the study protocol must also be provided in the manuscript.

Field-specific reporting

Please select the one below that is the best fit for your research. If you are not sure, read the appropriate sections before making your selection.

☒ Life sciences ☐ Behavioural & social sciences ☐ Ecological, evolutionary & environmental sciences

For a reference copy of the document with all sections, see nature.com/documents/nr-reporting-summary-flat.pdf

Life sciences study design

All studies must disclose on these points even when the disclosure is negative.

Sample size	NHANES data used in this study included all participants that were tested for MGUS. Individuals were sampled and tested by Landgren et al. (DOI: 10.1038/leu.2014.34) from the broader NHANES sample. Sample sizes were not relevant for SEER and CDC Wonder data as this included entire populations.
Data exclusions	For NHANES, samples that did not test for MGUS were excluded.
Replication	Each MCMC was repeated 5 times with a different seed, and convergence was assessed to ensure that the results were repeatable.
Randomization	Randomization was not relevant as individuals in the data used in this study were collected under observational studies.
Blinding	Blinding was not relevant as the data used in this study was collected under observation studies.

Reporting for specific materials, systems and methods

We require information from authors about some types of materials, experimental systems and methods used in many studies. Here, indicate whether each material, system or method listed is relevant to your study. If you are not sure if a list item applies to your research, read the appropriate section before selecting a response.

Materials & experimental systems

n/a	Involved in the study	
☒	☐	Antibodies
☒	☐	Eukaryotic cell lines
☒	☐	Palaeontology and archaeology
☒	☐	Animals and other organisms
☒	☐	Clinical data
☒	☐	Dual use research of concern
☒	☐	Plants

Methods

n/a	Involved in the study	
☒	☐	ChIP-seq
☒	☐	Flow cytometry
☒	☐	MRI-based neuroimaging
